# Trends and Characteristics of #HIVPrevention Tweets Posted Between 2014 and 2019: Retrospective Infodemiology Study

**DOI:** 10.2196/35937

**Published:** 2022-08-11

**Authors:** Raquel Burgess, Josemari T Feliciano, Leonardo Lizbinski, Yusuf Ransome

**Affiliations:** 1 Department of Social and Behavioral Sciences Yale School of Public Health Yale University New Haven, CT United States; 2 Department of Biostatistics Yale School of Public Health Yale University New Haven, CT United States; 3 The Warren Alpert Medical School Brown University Providence, RI United States

**Keywords:** HIV, social media, Twitter, prevention, infodemiology

## Abstract

**Background:**

Twitter is becoming an increasingly important avenue for people to seek information about HIV prevention. Tweets about HIV prevention may reflect or influence current norms about the acceptability of different HIV prevention methods. Therefore, it may be useful to empirically investigate trends in the level of attention paid to different HIV prevention topics on Twitter over time.

**Objective:**

The primary objective of this study was to investigate temporal trends in the frequency of tweets about different HIV prevention topics on Twitter between 2014 and 2019.

**Methods:**

We used the Twitter application programming interface to obtain English-language tweets employing #HIVPrevention between January 1, 2014, and December 31, 2019 (n=69,197, globally). Using iterative qualitative content analysis on samples of tweets, we developed a keyword list to categorize the tweets into 10 prevention topics (eg, condom use, preexposure prophylaxis [PrEP]) and compared the frequency of tweets mentioning each topic over time. We assessed the overall change in the proportions of #HIVPrevention tweets mentioning each prevention topic in 2019 as compared with 2014 using chi-square and Fisher exact tests. We also conducted descriptive analyses to identify the accounts posting the most original tweets, the accounts retweeted most frequently, the most frequently used word pairings, and the spatial distribution of tweets in the United States compared with the number of state-level HIV cases.

**Results:**

PrEP (13,895 tweets; 20.08% of all included tweets) and HIV testing (7688, 11.11%) were the most frequently mentioned topics, whereas condom use (2941, 4.25%) and postexposure prophylaxis (PEP; 823, 1.19%) were mentioned relatively less frequently. The proportions of tweets mentioning PrEP (327/2251, 14.53%, in 2014, 5067/12,971, 39.1%, in 2019; *P*≤.001), HIV testing (208/2251, 9.24%, in 2014, 2193/12,971, 16.91% in 2019; *P*≤.001), and PEP (25/2251, 1.11%, in 2014, 342/12,971, 2.64%, in 2019; *P*≤.001) were higher in 2019 compared with 2014, whereas the proportions of tweets mentioning abstinence, condom use, circumcision, harm reduction, and gender inequity were lower in 2019 compared with 2014. The top retweeted accounts were mostly UN-affiliated entities; celebrities and HIV advocates were also represented. Geotagged #HIVPrevention tweets in the United States between 2014 and 2019 (n=514) were positively correlated with the number of state-level HIV cases in 2019 (r=0.81, *P*≤.01).

**Conclusions:**

Twitter may be a useful source for identifying HIV prevention trends. During our evaluation period (2014-2019), the most frequently mentioned prevention topics were PrEP and HIV testing in tweets using #HIVPrevention. Strategic responses to these tweets that provide information about where to get tested or how to obtain PrEP may be potential approaches to reduce HIV incidence.

## Introduction

Globally, 1.5 million (1.1-2.0 million) people became infected with HIV in 2021 [1]. In the United States, an estimated 34,800 new HIV infections occurred in 2019, representing an 8% decline from 2015 [2]. An estimated 13% of HIV-infected individuals in the United States in 2019 did not know they were infected [3,4]. Levels of awareness of prevention methods such as preexposure prophylaxis (PrEP) are low among some high risk populations and there is substantial room for improvement in knowledge of HIV prevention across many states [5,6].

Social media sites are becoming increasingly important avenues for people of all age groups to seek information about health issues, including HIV [7]. Social media may be a particularly important avenue for promoting HIV prevention among younger people, given that younger people have a higher likelihood of using social media for health communication and they represent the highest burden of new HIV infections [2,8].

Previous research on social media and HIV information suggests that social media may be an effective avenue for spreading and consuming HIV information because it allows for anonymity and reduces stigma-related barriers to information seeking [7,9]. This may occur in part because discussing sexual health on social media mitigates the feelings of discomfort that can occur when discussing sexual health topics in-person among some population groups [7]. Yet, other research describes how a lack of privacy and the potential for bullying may deter individuals from sharing or interacting with sexual health content on social media [10,11].

Some research suggests that social media may have a beneficial effect on the adoption of HIV prevention behaviors. For example, social support provided by social media engagement prevention-specific messages have been associated with improved access to and uptake of HIV prevention and testing [7,12-15]. Moreover, interventions deployed via social media have been shown to increase HIV testing among men who have sex with men (MSM) [16] and to promote knowledge of sexually transmitted infections among young adults [17]. Support for the beneficial effect of HIV prevention communication on social media on HIV prevention behaviors is enhanced by evidence suggesting that higher rates of HIV-specific tweet activity per capita have been associated with lower HIV incidence in the following year [18].

Despite these positive findings, it has also been shown that Twitter can be used to propagate messages that perpetuate HIV-related stigma and endorse risky sexual behaviors [19]. These types of messages may also influence HIV incidence: in one study, the authors used an index of the proportion of Twitter users who posted risk behavior tweets (eg, “alcohol”, “without condom”) among all Twitter users to operationalize behavioral risk. They found that higher scores on the index were positively correlated with a higher rate of new HIV diagnoses across US counties [20]. Overall, this body of research suggests that social media messages about HIV can play an important role in HIV prevention and risk behavior.

Taggart and colleagues [21] further advance the view that “messaging matters” by providing a historical analysis of the evolution of public health messaging about HIV/AIDS. They provide evidence to suggest that initial public health communication about HIV was fear based, which transitioned to a focus on individual risk behaviors, and later, to empowerment and structural factors [21]. They also described the evolution of messaging about specific prevention methods. In the 1980s, HIV prevention messaging focused on harm reduction, such as safe sex and HIV testing. In response to innovations in HIV testing and treatment, messaging in the 2000s maintained focus on HIV testing while also promoting early detection and initiation of antiretroviral treatment. By the 2010s, PrEP was being promoted for high-risk individuals and later expanded to more general populations [21].

Importantly, Taggart and colleagues [21] described how messaging about prevention methods shifted social norms about the acceptability of HIV prevention methods over time. For example, PrEP was initially promoted only for individuals at a high risk of HIV, which may have contributed to PrEP-related stigma and slower-than-expected uptake of the drug [21]. Further research across other health issues corroborates the idea that social media can influence social norms about healthy behaviors, including with respect to sexual health [22-26].

This study was designed based on the same reasoning employed by Taggart and colleagues [21]: that is, there is utility in examining trends in how people talk about HIV prevention over time, as these trends may both reflect and influence changes in the acceptability and uptake of these prevention methods [13,16,27]. Trends in how individuals use social media to search for and provide health information can be studied using infodemiological approaches, which involve using information available on the internet to inform efforts to improve public health [28]. In this study, we used an infodemiological approach to examine temporal trends in the relative attention paid to different HIV prevention methods on Twitter. To the best of our knowledge, this is the first study to compare tweet activity about different HIV prevention topics and to investigate how tweet activity about HIV prevention topics has changed over time. Describing trends in the relative attention paid to different HIV prevention topics may provide public health professionals valuable insights about the acceptability and popularity of different HIV prevention methods; these insights could be used to inform strategic health communication efforts about HIV prevention.

We employed a passive, retrospective infodemiology approach in which we collected tweets that included #HIVPrevention (n=69,197) during a 6-year timeframe (2014-2019) corresponding to a critical period related to the uptake of PrEP in the United States and globally. We examined trends in the frequency of mentions of 10 different HIV prevention topics and assessed changes in the proportion of tweets mentioning each topic in 2019 as compared with 2014. We also report descriptive information on the spatial distribution of geotagged #HIVPrevention tweets in relation to the number of state-level HIV cases in the United States, the most frequently used word pairings in the tweets, the accounts posting the most original tweets, and the accounts retweeted most frequently. We conclude by discussing the implications of our findings and suggesting the opportunities for leveraging HIV prevention communication on Twitter to reduce HIV incidence.

## Methods

### Study Design

We conducted a retrospective infodemiology study using publicly available tweets employing #HIVPrevention between 2014 and 2019.

### Data

We utilized the Twitter application programming interface to collect all tweets (including original tweets, retweets, quote tweets, and replies) written in the English language that employed #HIVPrevention between January 1, 2014, and December 31, 2019 (n=69,197). We selected the timeframe 2014-2019 because it corresponds to a period following Food and Drug Administration (FDA) approval of PrEP for HIV prevention in the United States (occurring in 2012) [29]. Moreover, during this period, the World Health Organization (WHO) issued several expansions to its recommendations of population groups that should consider using PrEP [30]. Therefore, this is an interesting period to examine not only to understand changes in the attention paid to PrEP on Twitter, but also to understand how attention to other prevention methods may have changed during this time. We did not apply any geographical constraints to our sample as most tweets are not geotagged and individuals can be easily exposed to tweets generated in various regions of the world. We selected the hashtag #HIVPrevention as a proxy for HIV prevention-related tweets because it was the hashtag used by the Joint United Nations Programme on HIV/AIDS (UNAIDS) in 2016 to promote World AIDS Day (WAD) [31], and was used throughout the entire study period within tweets that discussed HIV prevention topics. WAD is an international day organized by UNAIDS to raise awareness about HIV [31]. In 2016, UNAIDS used #HIVPrevention to promote awareness of 9 different HIV prevention topics in the 9 weeks leading up to WAD; the topics were condoms, harm reduction, voluntary medical male circumcision (VMMC), elimination of mother-to-child transmission of HIV (EMTCT), PrEP, empowerment of young girls/women, testing viral suppression, targeting key populations, and investing in HIV prevention [31].

### Descriptive Analyses

We performed several descriptive analyses (eg, tabulation, Pearson correlation) to investigate the characteristics of the data. All analyses were conducted in R statistical software (version 4.0.3; R Foundation for Statistical Computing).

To investigate the change in activity related to tweets using #HIVPrevention over the study period, we tabulated original tweets (including replies and relevant quote tweets) and retweets (including relevant quote tweets) by month and year to identify trends.

To determine the Twitter accounts that generated the highest proportion of original #HIVPrevention tweets during the study period, we tabulated the number of original tweets as a function of unique account usernames.

To determine which Twitter accounts’ #HIVPrevention tweets were retweeted at the highest frequencies, we tabulated the number of retweets associated with #HIVPrevention tweets that each unique account username received.

To identify the most frequently used word pairings, also known as bigrams, we used the tidytext package (version 0.3.1) in R. This method allows for an indication of the context in which words are used. For example, a tweet containing the text “PrEP is an effective tool” will correspond to the following 2 bigrams: (1) PrEP and effective, and (2) effective and tool. Using *Gephi* (version 0.9.2), we created a visual word network of the top 50 bigrams found in our sample.

To understand the relationship between geotagged tweets and the number of HIV cases, we performed a Pearson correlation to assess the relationship between the number of geotagged tweets at the state level between 2014 and 2019 and the number of HIV cases in 2019 at the state level [32]. We mapped the geotagged tweets in the United States (n=514) and 2019 HIV cases at the state level using the leaflet package (version 2.0.4.1) in R.

### Analysis of the HIV Prevention Topics Referenced Most Often in #HIVPrevention Tweets

To determine the frequency at which various HIV prevention topics were mentioned in #HIVPrevention tweets and retweets and whether this changed over the study period, we first developed a list of 10 prevention topics and relevant keywords. We selected prevention topics based on the topics identified by the UNAIDS 2016 WAD campaign and our review of the literature. The 10 selected prevention topics were PrEP, postexposure prophylaxis (PEP), condom use, abstinence, VMMC, EMTCT, HIV testing, harm reduction, gender inequity and violence against women, and sex work.

We developed a keyword list for these 10 prevention topics by drawing on the initial stages of summative qualitative content analysis [33], in which text is explored to identify how words are used in context. For example, we were able to identify that the term “daily blue” was used to refer to PrEP without naming PrEP explicitly. We believe this process helped us to identify keywords that would have been otherwise overlooked and improved the accuracy of our tweet categorization.

**Figure 1:**
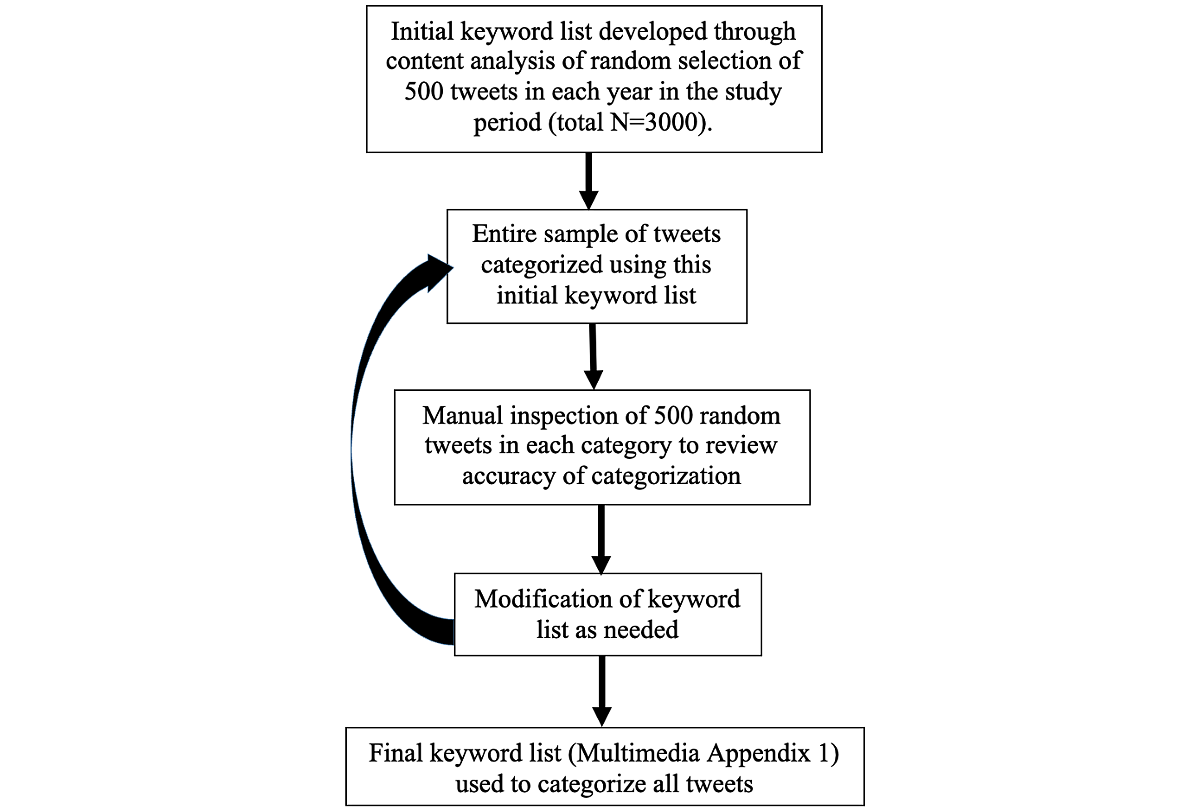
Manual inspection process for refining the keyword list.

Following initial development of our keyword list, we iteratively refined it using a manual inspection process to ensure that our keyword list had a high level of sensitivity and an acceptable level of specificity. That is, we sought to identify all tweets mentioning a particular prevention method (true positives) while minimizing any miscategorization (false positives). An example of a false positive would be a tweet referring to the US President’s Emergency Plan for AIDS Relief (PEPFAR) that was categorized under PEP. As some miscategorization was inevitable, we accepted an error level that was ≤5% (ie, in our manual inspection, ≤25 of the 500 inspected tweets were not related to the respective prevention method). If greater than 5% error was detected, we made appropriate modifications to our keyword list to fix the inaccuracies. We similarly inspected samples of the tweets which were uncategorized to determine if we missed any keywords that were relevant to a particular category (ie, to minimize false negatives). When these were discovered, we refined our keyword list to include the relevant keyword. If a tweet mentioned keywords related to more than 1 prevention topic (eg, “PrEP”, “condom”), then that tweet was categorized in each respective category. If a tweet mentioned multiple keywords related to the same prevention category, that tweet was counted in the respective category only once. The manual inspection process was conducted by the first author (RB) and the final list of keywords (Multimedia Appendix 1) was further verified by the senior author (YR), a content expert in HIV. A depiction of our iterative manual inspection process for refining our keyword list is presented in Figure 1.

To evaluate how attention to each topic changed over the study period, we compared the proportion of tweets related to each respective topic in 2019 with the proportion of tweets related to each respective topic in 2014 using chi-square and Fisher exact tests.

### Ethical Considerations

The study was granted an Ethics Exemption by the Yale University Institutional Review Board (#2000028381).

## Results

### Descriptive Analyses

Our sample consisted of 25,031 original tweets and 44,166 retweets, totaling 69,197 tweets. Geotagged tweets represented 1.81% (n=1253) of the sample and were tweeted from 76 countries.

**Figure 2:**
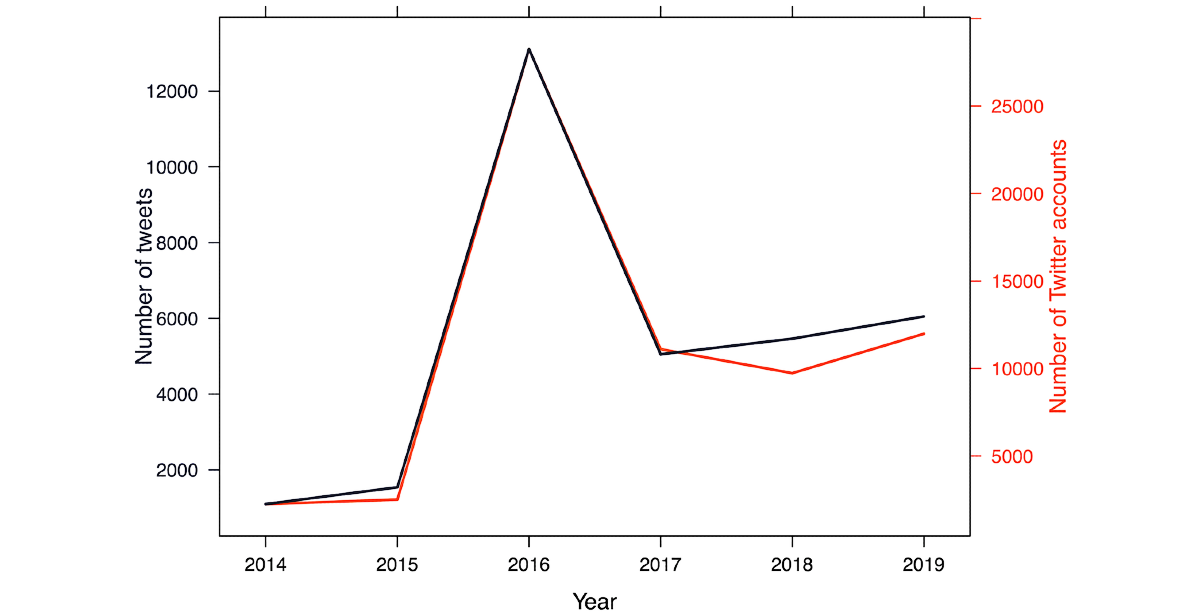
Annual frequency of #HIVPrevention tweets between 2014 and 2019.

Figure 2 presents the number of tweets (including retweets) and unique accounts employing #HIVPrevention between 2014 and 2019 as a function of the year. The number of tweets and accounts using #HIVPrevention is lowest in 2014, at 2251/69,197 tweets (3.25% of the total sample) generated by 1097 accounts. A substantial increase in tweet activity is observed in 2016 as compared with 2014 and 2015, with 28,254/69,197 tweets (40.83%) posted in 2016 from 13,109 accounts. A closer inspection of the data confirmed that the 2016 tweets were primarily related to WAD. This substantial increase in activity was not sustained in 2017 (10,811/69,197 tweets, 15.62%; 5188 accounts), although the number of #HIVPrevention tweets posted in 2017 was higher than the number posted in 2015 (3209/69,197 tweets, 4.64%; 1215 accounts). This likely reflects higher usage of the term #HIVPrevention following the 2016 campaign rather than an actual increase in the number of tweets addressing HIV prevention, although it could reflect both factors. The number of users utilizing #HIVPrevention follows a similar pattern to the number of total tweets over the study period.

The 10 accounts that generated the most original #HIVPrevention tweets between 2014 and 2019 are presented in Table 1. The individual who is responsible for the account @HIV_Insight also reports being responsible for @Sex_Worker_Hlth and @Hlth_Literacy, suggesting that they are responsible for 66.37% (4072/6135) of the original content provided by the top 10 content contributing accounts. @DrMbere, @Health_HIV2030, and @himmoderator also identify as individual-run accounts in their Twitter account descriptions, whereas the remaining accounts were identified as run by institutions. In total, individuals are responsible for 80.84% (4960/6135) of the original content provided by the top 10 content contributing accounts.

**Table 1:** Accounts with the most original and retweeted #HIVPrevention tweets between 2014 and 2019.

Accounts with the most original #HIVPrevention tweets	Number of original tweets per account	Accounts whose #HIVPrevention tweets were retweeted at the highest frequencies	Number of retweets per account
@HIV_Insight	3144	@UNAIDS	11,239
@Sex_Worker_Hlth	484	@HIV_Insight	1880
@DrMbere	465	@MichelSidibe	1551
@Hlth_Literacy	444	@UN	908
@HIVIreland	396	@MissUniverse	705
@UNAIDS	296	@UNAIDS_AP	687
@EPICBrowardOrg	262	@HIVpxresearch	499
@Health_HIV2030	240	@accphivprn	493
@HopeandHelpInc	221	@AniShakari	470
@himmoderator	183	@HIVIreland	468

The 10 accounts whose #HIVPrevention tweets were retweeted at the highest frequencies between 2014 and 2019 are presented in Table 1. The United Nations is responsible for 3 of these accounts (@UN, @UNAIDS, and @UNAIDS_AP [UNAIDS Asia-Pacific]) and the accounts @MichelSidebe and @AniShakari publicly identify themselves as current or former employees of UNAIDS. The remaining accounts are also run by institutions that work on the HIV/AIDS epidemic, with the notable exceptions of @HIV_Insight and @MissUniverse; the latter ran a campaign on HIV prevention in 2016 for WAD, which involved promotion of various HIV prevention methods by Miss Universe contestants. The accounts @UN (12,047,848 followers), @MissUniverse (1,022,563), and @UNAIDS (258,322) corresponded to the largest numbers of followers at the time of data collection (March 2020).

**Figure 3:**
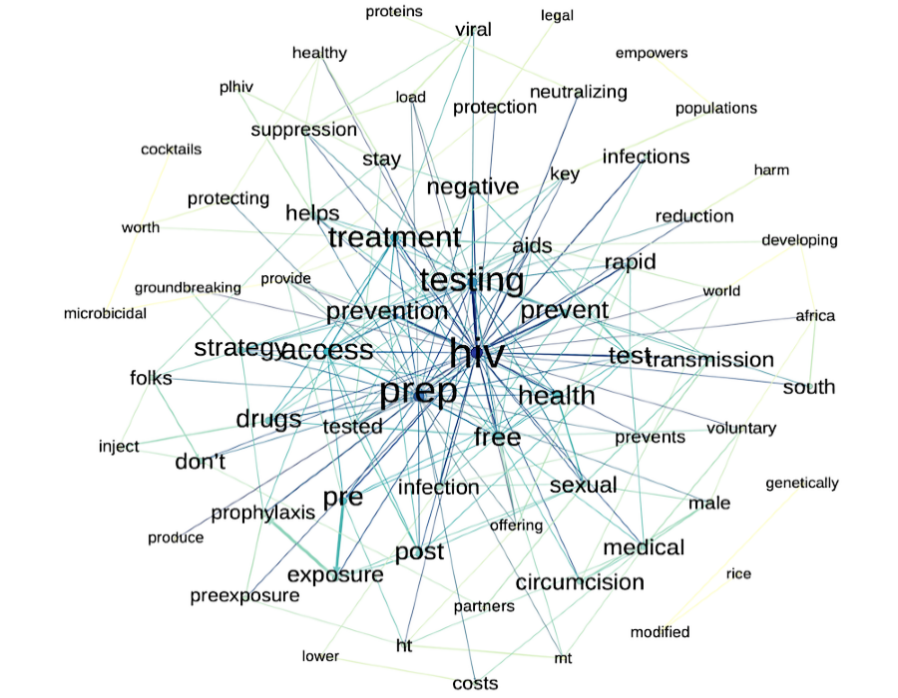
A visual word network of the 50 most frequently used bigrams (word-pairings) in #HIVPrevention tweets between 2014 and 2019.

Figure 3 presents a word network depicting the 50 most frequently used bigrams seen in #HIVPrevention tweets between 2014 and 2019. The most frequently used words (not listed in the order of frequency) were PrEP, testing, treatment, prevent, access, strategy, transmission, HIV, health, and free.

**Figure 4:**
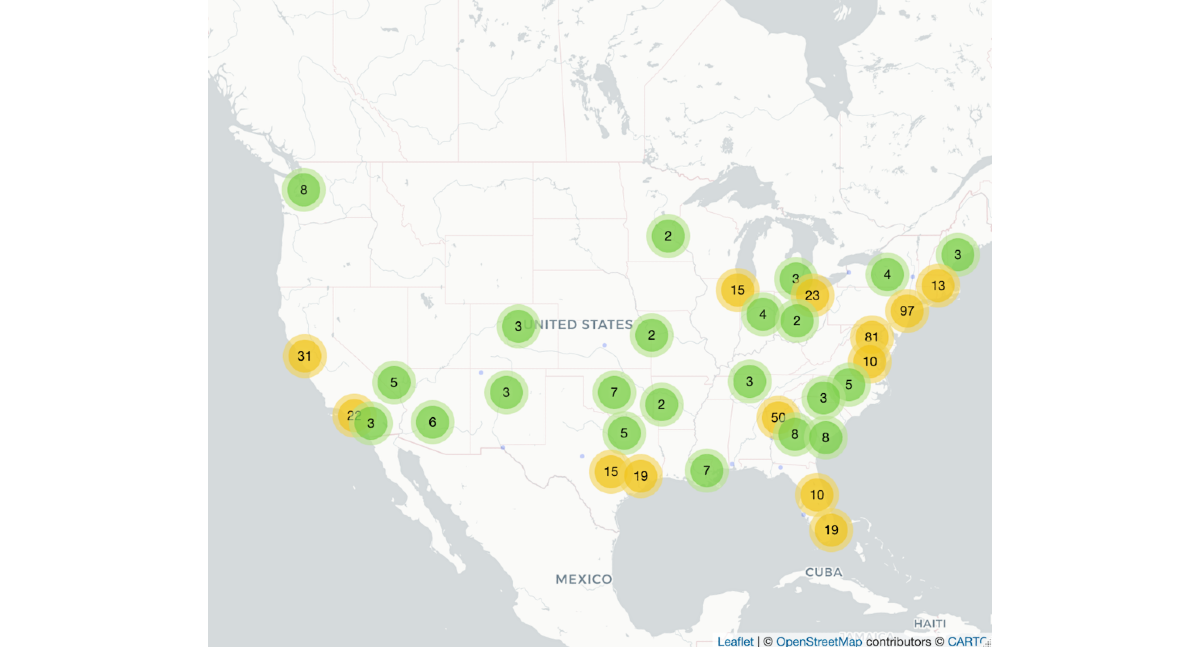
Geographic distribution of geotagged English #HIVPrevention tweets (n=514) in the United States between 2014 and 2019. The numbers in the figure correspond to the number of tweets geotagged to the respective locations indicated on the map. The mapping data presented here is available under the Open Database (CC-BY-SA) License [34].

**Figure 5:**
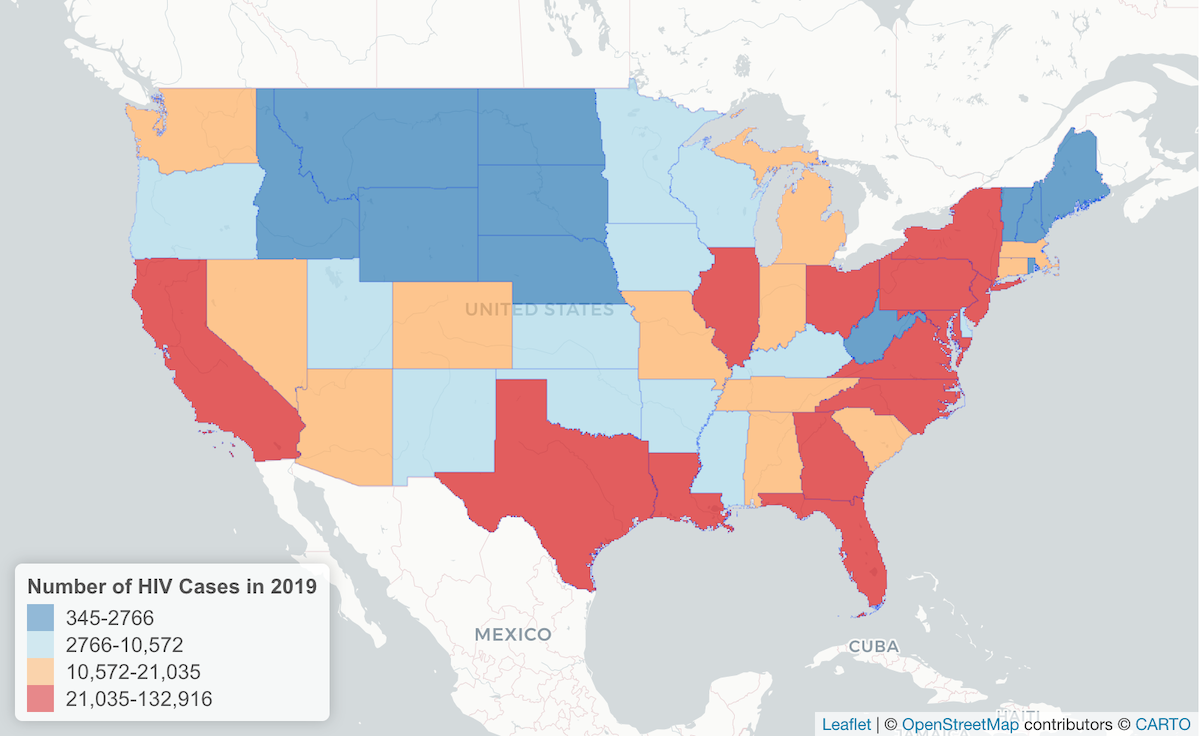
Geographic distribution of the number of HIV cases in the United States in 2019, displayed at the state level. The mapping data presented here is available under the Open Database (CC-BY-SA) License [34].

**Figure 6:**
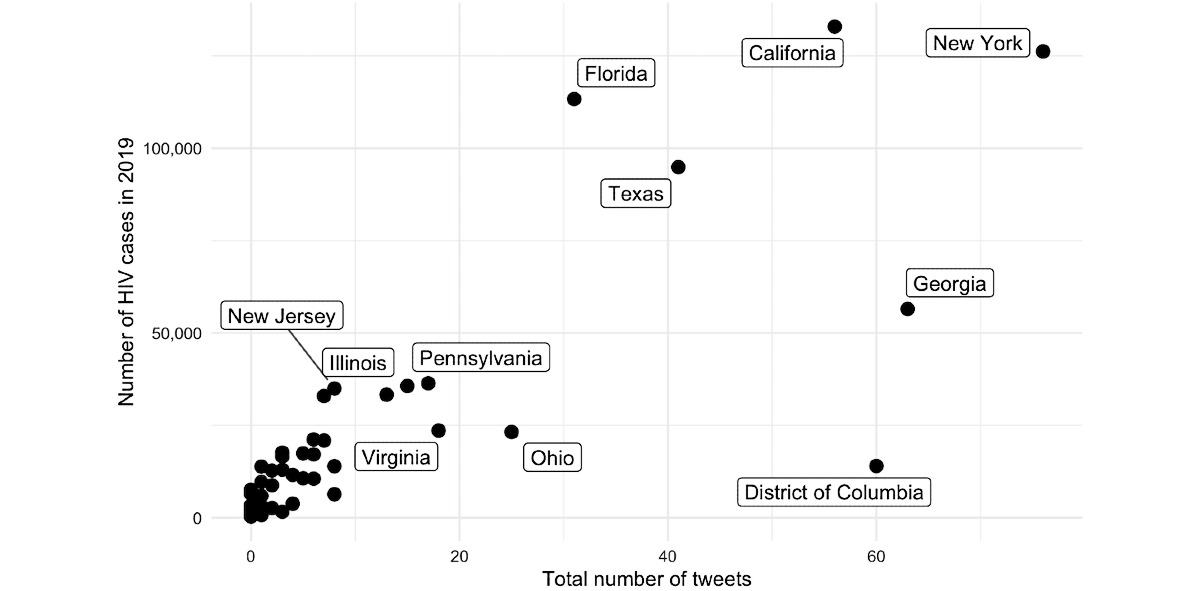
Relationship between the total number of geotagged #HIVPrevention tweets at the state level between 2014-2019 and the number of 2019 HIV cases by state.

Figures 4 and 5 display the geographic distribution of geotagged #HIVPrevention tweets between 2014 and 2019 and the number of state-level HIV cases in 2019, respectively. The data set presented in Figure 5 is publicly available from the Centers of Disease Control and Prevention [32]. The number of #HIVPrevention tweets per state was positively correlated with the number of state-level HIV cases in 2019 (*r*=0.81, *P*<.01; Figure 6).

### Analysis of the HIV Prevention Topics Being Referenced Most Often in #HIVPrevention Tweets

Of the total 69,197 #HIVPrevention tweets in the sample, 28,135 tweets (40.66%) were categorized into 1 or more of the 10 identified prevention topics. The highest proportion of mentions were seen for PrEP (13,895/69,197 tweets, 20.08% of all tweets). This was followed by the proportion of mentions related to HIV testing (7688/69,197, 11.11%), condoms (2941/69,197, 4.25%), harm reduction (2173/69,197, 3.14%), gender equity and violence against women (1695/69,197, 2.45%), VMMC (969/69,197, 1.40%), sex work (872/69,197, 1.26%), PEP (823/69,197, 1.19%), EMTCT (277/69,197, 0.40%), and abstinence (180/69,197, 0.26%). Categorized tweet totals do not add to 28,135, given that some tweets were categorized in more than 1 category.

**Figure 7:**
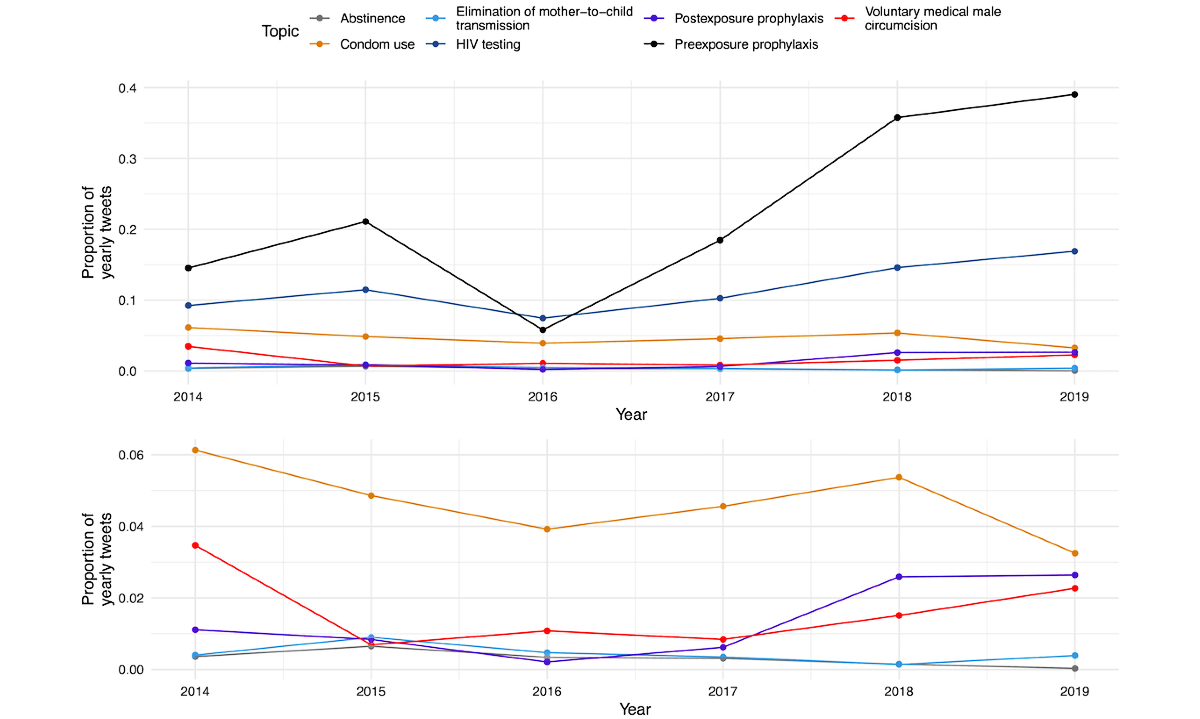
Annual frequency of mentions of keywords related to abstinence, condom use, elimination of mother-to-child transmission, HIV testing, post-exposure prophylaxis, pre-exposure prophylaxis, and voluntary medical male circumcision as a proportion of total annual #HIVPrevention tweets between 2014 and 2019.

Figure 7 illustrates the proportion of annual topic-specific tweets (original and retweets) as a function of total annual tweets for the following direct prevention topics: abstinence, condoms, PEP, testing, VMMC, EMTCT, and PrEP. The bottom panel of Figure 7 is presented on a smaller scale so the reader can better see the trends in the frequency of mentions of condom use, VMMC, PEP, EMTCT, and abstinence.

Table 2 displays the results of our chi-square and Fisher exact tests. The proportion of tweets mentioning PrEP, HIV testing, and PEP significantly increased between 2014 and 2019 (*P*≤.01 for all cases), whereas the proportion of tweets mentioning abstinence, condom use, VMMC, harm reduction, and gender equity significantly decreased in this period (*P*≤.01 for all cases).

**Table 2:** Results of the chi-square and Fisher exact tests^a^ evaluating the overall change in the proportion of tweets related to each topic area in 2014 versus 2019.

Prevention topic	2014 tweets (n=2251), n (%)	2019 tweets (n=12,971), n (%)	*P* value	Direction of change
Abstinence	8 (0.36)	4 (0.03)	≤*.01*^b^	Lower
Condom use	138 (6.13)	421 (3.25)	≤*.01*^b^	Lower
Preexposure prophylaxis	327 (14.53)	5067 (39.06)	≤*.01*^b^	Higher
Voluntary medical male circumcision	78 (3.47)	295 (2.27)	≤*.01*^b^	Lower
Postexposure prophylaxis	25 (1.11)	342 (2.64)	≤*.01*^b^	Higher
Harm reduction	93 (4.13)	336 (2.59)	≤*.01*^b^	Lower
Gender inequity and violence against women	74 (3.29)	251 (1.94)	≤*.01*^b^	Lower
Elimination of mother-to-child transmission	9 (0.40)	51 (0.39)	.96	N/A^c^
Sex work	39 (1.73)	198 (1.53)	.47	N/A
HIV testing	208 (9.24)	2193 (16.91)	≤*.01*	Higher

^a^Fisher exact test used when expected frequencies are less than 5.

^b^Italicized values are statistically significant.

^c^N/A: not applicable.

## Discussion

### Principal Findings

In this study, we investigated temporal trends in the frequency of mentions of 10 different HIV prevention topics in #HIVPrevention tweets between 2014 and 2019. Our findings describe how attention to different HIV prevention methods on Twitter has changed over time, which may provide insight into changes in the acceptability and uptake of these prevention methods. We also report useful descriptive information about our sample, such as the characteristics of accounts receiving the most retweets of #HIVPrevention tweets. These findings may assist public health professionals in identifying strategic approaches to improving the dissemination of HIV prevention information on Twitter.

Key findings from our analysis include the following: both PrEP and HIV testing were discussed at relatively high frequencies during the study period as compared with other HIV prevention methods such as condom use, VMMC, EMTCT, and PEP. Moreover, there were significantly higher proportions of #HIVPrevention tweets mentioning PrEP, HIV testing, and PEP in 2019 as compared with 2014, although the largest changes are seen for PrEP and testing. There were significantly lower proportions of #HIVPrevention tweets mentioning abstinence, VMMC, condom use, harm reduction, and gender inequity and violence against women in 2019 as compared with 2014. The increases in the proportion of tweets related to PrEP in 2017, 2018, and 2019 likely reflect approvals of PrEP for use in countries around the world between 2016 and 2018, including South Africa, South Korea, and the European Union [35], and updates to the WHO recommendations on which populations should use PrEP [30]. The relatively lower proportion of PrEP-related tweets in 2016 may reflect a larger representation of other prevention methods in comparison to PrEP during the WAD campaign. The significant decrease in the proportion of tweets mentioning condom use in 2019 as compared with 2014 might reflect the decrease in condom use which has been associated with uptake of PrEP in some populations [36,37]. The decline in abstinence-related tweets likely reflects the shift away from limited efficacy abstinence-based approaches to HIV prevention [38].

The high proportion of #HIVPrevention tweets related to PrEP and HIV testing is promising given that PrEP is highly effective at preventing HIV transmission [39] and research suggests that social media messages about PrEP directly or indirectly (through communication about PrEP use) correlate with HIV testing and PrEP use in regions with higher populations of MSM [12]. Moreover, a recent modeling study found that a scale-up of targeted PrEP and HIV testing in 6 US cities is expected to yield the largest reduction in new HIV infections as compared with other strategies such as harm reduction and initiation and retention of individuals diagnosed with HIV in antiretroviral therapy [40].

However, optimal adherence to PrEP can be a challenge for at-risk individuals; barriers include stigma, health system inaccessibility, and competing life stressors [41]. The efficacy of PrEP decreases as adherence decreases and with 2 doses per week, PrEP efficacy is similar to that of consistent condom use [39,42]. Moreover, the cost of PrEP (estimated at around US $24,000 a year) may pose a barrier to uptake by populations who do not have health insurance; even with insurance, out-of-pocket costs can be substantial [43]. For some populations, strong advocacy surrounding PrEP use for HIV prevention may deflect attention from more economically feasible or individually preferred prevention methods such as condoms [40,44]. It may also divert attention from the underlying social determinants of health relevant to HIV such as addiction, sex work, poverty, racial inequities, and gender inequity [13]; both gender equity and harm reduction represented proportionally less #HIVPrevention tweets (although higher absolute numbers) in 2019 compared with 2014. Scholars suggest that a combination of prevention options is required to effectively combat the HIV epidemic [22], as any one prevention method is unlikely to be a panacea. Our analysis suggests that condom use and especially PEP, EMTCT, and VMMC have received relatively little attention compared with PrEP on this platform; it may prove advantageous to ensure that information about these prevention topics is disseminated widely on Twitter to increase uptake and acceptability [5,40].

### Secondary Findings

Although our analysis reveals that individuals are responsible for the majority of accounts that correspond to the highest number of original #HIVPrevention tweets, UN-affiliated institutions and individuals appear to be reaching the most people as indicated by their retweet and follower counts, an unsurprising finding given the 2016 WAD campaign. However, the analysis of content generating and retweeted accounts also reveals the importance of informal advocates (eg, @HIV_Insight) and celebrity endorsements (eg, @MissUniverse); the latter may be particularly effective given the sheer number of users following celebrity accounts and the influence celebrities can have on health promotion [45].

Finally, our analysis of geotagged tweets suggests that #HIVPrevention tweets at a state level between 2014 and 2019 are positively correlated with the number of state-level HIV cases in the United States in 2019. This finding should be interpreted with caution given the small number of tweets in our sample that were geotagged. However, this finding is aligned with other research that suggests that tweet content such as discussing HIV risk-related behavior (eg, drug use) is associated with the geographic distribution of HIV [18,20].

### Study Strengths and Implications

To the best of our knowledge, this study is the first to investigate temporal trends in the relative attention received by different HIV prevention methods on Twitter. We are hopeful that the findings provide useful insight into how attention to HIV prevention methods on Twitter has changed over time, which may reflect or influence changes in the acceptability of these methods.

Some of the findings may be useful in informing strategic approaches to the dissemination of HIV prevention information on Twitter. For example, the findings indicate that a large portion of #HIVPrevention tweets mention PrEP and HIV testing. These tweets could be responded to by providing specific information about where to obtain an HIV test or how to access PrEP, which may empower individuals to engage in these behaviors. Furthermore, public health entities could consider leveraging celebrities as HIV advocates on Twitter given their wide reach and popularity, especially with young people. Finally, public health institutions may consider increasing communication about certain HIV prevention methods on Twitter such as condom use to ensure that populations with diverse needs and resources are aware of the HIV prevention options available to them.

### Study Limitations

There are some limitations of our study. First, we were limited to results procured from a single Twitter hashtag. Although this was a necessary methodological decision to define a sample of tweets focused on HIV prevention, it omits tweets that discuss HIV prevention but do not employ #HIVPrevention and it is possible that these tweets differ importantly from those that do employ the hashtag. Although we examined tweets corresponding to a critical period in the evolution of the acceptability of PrEP, resource and feasibility constraints limited us from investigating tweets posted immediately after PrEP was approved in the United States in 2012. We point the reader to previous research that yields insights into earlier periods [46-48] and encourage other researchers to investigate HIV prevention discussion on Twitter during the COVID-19 pandemic. Some prevention topics, such as gender inequity and harm reduction, are relatively more abstract and difficult to capture than others (eg, PrEP). Moreover, our analysis was restricted to tweets that are written in English, which obscures insights about HIV prevention discussions in other languages. This limits the validity of our map of geotagged #HIVPrevention tweets as a marker of overall discussion about HIV prevention on Twitter in the United States, as does the relatively small proportion of #HIVPrevention tweets that were geotagged. Moreover, disparities in access to internet services across the United States [49] may have influenced the characteristics of individuals who were tweeting about HIV prevention in our sample and the regions the tweets originated from. We were unable to ascertain the age or other demographic characteristics of the users in our data as a variable in our analysis.

### Conclusions

Twitter is an important avenue for information seeking about HIV prevention and may be a particularly important platform for disseminating information to young adults who represent a large burden of new infections [2,50]. Previous evidence suggests that public health messaging shapes the ways in which we conceptualize and respond to the HIV epidemic and thus examining trends in communication about HIV prevention over time is an important step for better understanding the course of the epidemic and planning effective strategies for the future [21]. The findings of our study indicate that PrEP and HIV testing have received the most attention in #HIVPrevention tweets between 2014 and 2019 as compared with other HIV prevention topics and that attention to PrEP and HIV testing in #HIVPrevention tweets increased over that period. Public health professionals may wish to leverage the findings to inform multifaceted efforts toward reducing HIV incidence.

## Appendix

Multimedia Appendix 1 Identified prevention methods, associated keywords and examples of tweets related to each topic.

## Abbreviations

EHE: ending the HIV epidemic in the United States
EMTCT: elimination of mother-to-child transmission
FDA: Food and Drug Administration
MSM: men who have sex with men
PEP: postexposure prophylaxis
PEPFAR: US President’s Emergency Plan for AIDS Relief
PrEP: preexposure prophylaxis
UNAIDS: Joint United Nations Programme on HIV/AIDS
VMMC: voluntary medical male circumcision
WAD: World AIDS Day
WHO: World Health Organization

## Data Availability

Data from this study are available upon request. Please email requests to josemari.feliciano@yale.edu. Proof of ethics approval/exemption for proposed use may be required.

## References

[1] UNAIDS.

[2] (2021). Estimated HIV incidence and prevalence in the United States 2015-2019: HIV Surveillance Supplemental Report. CDC.

[3] Basic Statistics. Centers for Disease Control and Prevention.

[4] (2019). HIV Surveillance Report. Centers for Disease Control and Prevention.

[5] Koblin BA, Usher D, Nandi V, Tieu H, Bravo E, Lucy D, Miles L, Ortiz G, Kindlon MJ, Parisi DM, Frye V (2018). Post-exposure Prophylaxis Awareness, Knowledge, Access and Use Among Three Populations in New York City, 2016-17. AIDS Behav.

[6] Eaton LA, Matthews DD, Driffin DD, Bukowski L, Wilson PA, Stall RD, POWER Study Team (2017). A Multi-US City Assessment of Awareness and Uptake of Pre-exposure Prophylaxis (PrEP) for HIV Prevention Among Black Men and Transgender Women Who Have Sex with Men. Prev Sci.

[7] Taggart T, Grewe M, Conserve D, Gliwa C, Roman Isler Malika (2015). Social Media and HIV: A Systematic Review of Uses of Social Media in HIV Communication. J Med Internet Res.

[8] Huo J, Desai R, Hong Y, Turner K, Mainous A, Bian J (2019). Use of Social Media in Health Communication: Findings From the Health Information National Trends Survey 2013, 2014, and 2017. Cancer Control.

[9] Ko N, Hsieh C, Wang M, Lee C, Chen C, Chung A, Hsu S (2013). Effects of Internet popular opinion leaders (iPOL) among Internet-using men who have sex with men. J Med Internet Res.

[10] Byron P, Albury K, Evers C (2013). “It would be weird to have that on Facebook”: young people's use of social media and the risk of sharing sexual health information. Reproductive Health Matters.

[11] Kesten J, Dias K, Burns F, Crook P, Howarth A, Mercer C, Rodger A, Simms I, Oliver I, Hickman M, Hughes G, Weatherburn P (2019). Acceptability and potential impact of delivering sexual health promotion information through social media and dating apps to MSM in England: a qualitative study. BMC Public Health.

[12] Chan M, Morales A, Zlotorzynska M, Sullivan P, Sanchez T, Zhai C, Albarracín Dolores (2021). Estimating the influence of Twitter on pre-exposure prophylaxis use and HIV testing as a function of rates of men who have sex with men in the United States. AIDS.

[13] León Agathe, Cáceres César, Fernández Emma, Chausa P, Martin M, Codina C, Rousaud A, Blanch J, Mallolas J, Martinez E, Blanco JL, Laguno M, Larrousse M, Milinkovic A, Zamora L, Canal N, Miró Josep M, Gatell JM, Gómez Enrique J, García Felipe (2011). A new multidisciplinary home care telemedicine system to monitor stable chronic human immunodeficiency virus-infected patients: a randomized study. PLoS One.

[14] Hailey J, Arscott J (2013). Using technology to effectively engage adolescents and young adults into care: STAR TRACK Adherence Program. J Assoc Nurses AIDS Care.

[15] Mo PKH, Coulson NS (2008). Exploring the communication of social support within virtual communities: a content analysis of messages posted to an online HIV/AIDS support group. Cyberpsychol Behav.

[16] Cao B, Gupta S, Wang J, Hightow-Weidman L, Muessig K, Tang W, Pan S, Pendse R, Tucker J (2017). Social Media Interventions to Promote HIV Testing, Linkage, Adherence, and Retention: Systematic Review and Meta-Analysis. J Med Internet Res.

[17] Jones K, Eathington P, Baldwin K, Sipsma H (2014). The impact of health education transmitted via social media or text messaging on adolescent and young adult risky sexual behavior: a systematic review of the literature. Sex Transm Dis.

[18] Stevens R, Bonett S, Bannon J, Chittamuru D, Slaff B, Browne SK, Huang S, Bauermeister JA (2020). Association Between HIV-Related Tweets and HIV Incidence in the United States: Infodemiology Study. J Med Internet Res.

[19] Roszkowska N, Lazarus E, Bannon J, Dowshen N, Stevens R (2020). 2. From Bots to Jokes: Is There a Place For HIV Prevention on Twitter?. Journal of Adolescent Health.

[20] Li Z, Qiao S, Jiang Y, Li X (2021). Building a social media-based HIV risk behavior index to inform the prediction of HIV new diagnosis: a feasibility study. AIDS.

[21] Taggart T, Ritchwood TD, Nyhan K, Ransome Y (2021). Messaging matters: achieving equity in the HIV response through public health communication. The Lancet HIV.

[22] Litt D, Rodriguez L, Stewart S (2021). Examining Associations Between Social Networking Site Alcohol-Specific Social Norms, Posting Behavior, and Drinking to Cope During the COVID-19 Pandemic. Cyberpsychol Behav Soc Netw.

[23] Young S, Jordan A (2013). The influence of social networking photos on social norms and sexual health behaviors. Cyberpsychol Behav Soc Netw.

[24] Cerezo A, Ramirez A, O'Shaughnessy Tiffany, Sanchez A, Mattis S, Ross A (2021). Understanding the Power of Social Media during COVID-19: Forming Social Norms for Drinking among Sexual Minority Gender Expansive College Women. J Homosex.

[25] Lee T, Su L (2020). When a Personal HPV Story on a Blog Influences Perceived Social Norms: The Roles of Personal Experience, Framing, Perceived Similarity, and Social Media Metrics. Health Commun.

[26] Wombacher K, Reno J, Veil S (2017). NekNominate: Social Norms, Social Media, and Binge Drinking. Health Commun.

[27] Storey D, Seifert-Ahanda K, Andaluz A, Tsoi B, Matsuki J, Cutler B (2014). What is health communication and how does it affect the HIV/AIDS continuum of care? A brief primer and case study from New York City. J Acquir Immune Defic Syndr.

[28] Eysenbach G (2009). Infodemiology and infoveillance: framework for an emerging set of public health informatics methods to analyze search, communication and publication behavior on the Internet. J Med Internet Res.

[29] (2019). In Brief: FDA continues to encourage ongoing education about the benefits and risks associated with PrEP, including additional steps to help reduce the risk of getting HIV. Food and Drug Administration (FDA).

[30] (2015). WHO Expands Recommendation on Oral Pre-Exposure Prophylaxis of HIV Infection (PrEP). World Health Organization (WHO).

[31] (2016). World AIDS Day 2016. UNAIDS.

[32] NCHHSTP Atlas Plus. Centers for Disease Control and Prevention (CDC).

[33] Hsieh H, Shannon SE (2005). Three approaches to qualitative content analysis. Qual Health Res.

[34] OpenStreetMap.

[35] (2021). National Policies and Guidelines for PrEP. PrEPWatch.

[36] Holt M, Lea T, Mao L, Kolstee J, Zablotska I, Duck T, Allan B, West M, Lee E, Hull P, Grulich A, De Wit J, Prestage G (2018). Community-level changes in condom use and uptake of HIV pre-exposure prophylaxis by gay and bisexual men in Melbourne and Sydney, Australia: results of repeated behavioural surveillance in 2013-17. Lancet HIV.

[37] Montaño Michalina A, Dombrowski JC, Dasgupta S, Golden MR, Duerr A, Manhart LE, Barbee LA, Khosropour CM (2019). Changes in Sexual Behavior and STI Diagnoses Among MSM Initiating PrEP in a Clinic Setting. AIDS Behav.

[38] Santelli J, Speizer I, Edelstein Z (2013). Abstinence promotion under PEPFAR: the shifting focus of HIV prevention for youth. Glob Public Health.

[39] Anderson PL, Glidden DV, Liu A, Buchbinder S, Lama JR, Guanira JV, McMahan V, Bushman LR, Casapía Martín, Montoya-Herrera O, Veloso VG, Mayer KH, Chariyalertsak S, Schechter M, Bekker L, Kallás Esper Georges, Grant RM, iPrEx Study Team (2012). Emtricitabine-tenofovir concentrations and pre-exposure prophylaxis efficacy in men who have sex with men. Sci Transl Med.

[40] Krebs E, Dale L (2020). The impact of localized implementation: determining the cost-effectiveness of HIV prevention and care interventions across six United States cities. HIV Spec.

[41] Wood S, Gross R, Shea JA, Bauermeister JA, Franklin J, Petsis D, Swyryn M, Lalley-Chareczko L, Koenig HC, Dowshen N (2019). Barriers and Facilitators of PrEP Adherence for Young Men and Transgender Women of Color. AIDS Behav.

[42] Weller S, Davis-Beaty K (2002). Using condoms consistently reduces sexual transmission of HIV infection. Cochrane.

[43] Furukawa NW, Zhu W, Huang YA, Shrestha RK, Hoover KW (2020). National Trends in Drug Payments for HIV Preexposure Prophylaxis in the United States, 2014 to 2018. Ann Intern Med.

[44] Greene GJ, Swann G, Fought AJ, Carballo-Diéguez Alex, Hope TJ, Kiser PF, Mustanski B, D'Aquila Richard T (2017). Preferences for Long-Acting Pre-exposure Prophylaxis (PrEP), Daily Oral PrEP, or Condoms for HIV Prevention Among U.S. Men Who Have Sex with Men. AIDS Behav.

[45] Francis DB, Stevens EM, Noar SM, Widman L (2018). Public Reactions to and Impact of Celebrity Health Announcements: Understanding the Charlie Sheen Effect. Howard Journal of Communications.

[46] Odlum M, Yoon S, Broadwell P, Brewer R, Kuang D (2018). How Twitter Can Support the HIV/AIDS Response to Achieve the 2030 Eradication Goal: In-Depth Thematic Analysis of World AIDS Day Tweets. JMIR Public Health Surveill.

[47] McLaughlin ML, Hou J, Meng J, Hu C, An Z, Park M, Nam Y (2016). Propagation of Information About Preexposure Prophylaxis (PrEP) for HIV Prevention Through Twitter. Health Commun.

[48] Schwartz J, Grimm J (2017). PrEP on Twitter: Information, Barriers, and Stigma. Health Commun.

[49] Yu RP, Ellison NB, McCammon RJ, Langa KM (2015). Mapping the two levels of digital divide: Internet access and social network site adoption among older adults in the USA. Information, Communication & Society.

[50] Lim MS, Vella A, Sacks-Davis R, Hellard ME (2014). Young people's comfort receiving sexual health information via social media and other sources. Int J STD AIDS.

