# Supplementary material for: Trends and Characteristics of #HIVPrevention Tweets Posted Between 2014 and 2019: Retrospective Infodemiology Study
Source: JMIR Public Health Surveill. 2022 Aug 11;8(8):e35937. doi: 10.2196/35937 (PMC9412898; doi:10.2196/35937)
Supplement: Multimedia Appendix 1 [file publichealth_v8i8e35937_app1.docx]

**Appendix Table 1.** Identified prevention methods, associated keywords and examples of tweets related to each topic.^a,b^

| Prevention Topic | Keywords | Example Tweet (Year)^c^ |
| --- | --- | --- |
| Pre-exposure prophylaxis (PrEP) | Pre-exposure prophylaxis | 'PrEP is the biggest breakthrough in HIV management in decades' – [Name omitted] #hivprevention #PrEP (2016) |
|  | Preexposure prophylaxis |  |
|  | Pre-exposure |  |
|  | Preexposure |  |
|  | PrEP |  |
|  | Truvada |  |
|  | Prevention pill |  |
|  | Daily blue |  |
|  | Descovy |  |
| Post-exposure prophylaxis (PEP) | Post-exposure prophylaxis | People who are victims of rape or sexual assault can benefit from PEP use to lower risk of contracting HIV #PePTalk #HIVPrevention (2014) |
|  | Postexposure prophylaxis |  |
|  | Post-exposure prevention |  |
|  | Postexposure prevention |  |
|  | Post-exposure |  |
|  | postexposure |  |
|  | PEP |  |
|  | *Removed: PEPFAR* |  |
| Condom use | Condom(s) | Female condoms are estimated to reduce risk of HIV infection by 97.1% when used consistently and correctly #HIVPrevention (2014) |
|  | ABC |  |
|  | Wrap it up |  |
|  | #dontflexwithoutlatex |  |
|  | #don’tflexwithoutlatex |  |
| Abstinence | Abstinence | Abstinence remains the best way to #preventhiv #WorldAIDSDay  #hivprevention (2016) |
|  | ABCs |  |
|  | abstain |  |
| Voluntary medical male circumcision (VMMC) | Circumcision | Complications rare with baby #circumcisions, but they rise with age #HIVprevention (2014) |
|  | Circumcised |  |
|  | VMMC |  |
|  | MMC |  |
|  | #malecirc |  |
| Elimination of mother-to-child-transmission (EMTCT) | EMTCT | PEOPLE LIVING WITH HIV CAN STILL HAVE HEALTHY, HIV-NEGATIVE CHILDREN #HIVawareness #HIVprevention #PMTCT...(2015) |
|  | PMTCT |  |
|  | MTCT |  |
|  | PPTCT |  |
|  | Mother to child |  |
|  | Mother-to-child |  |
|  | Vertical transmission |  |
| HIV Testing | Test  Know your status | It is much better to #KnowYourStatus so that if your #HIV positive you can seek treatment and get back to living a happy and cool life and if your negative so that you can still take on #HIVprevention as a priority #KnowYourStatus #worlds2018 (2018) |
|  | Knowing your status |  |
|  | Know their status |  |
|  | Knowing their status |  |
|  | Knowing your #HIV status |  |
|  | Get checked |  |
|  | Screening |  |
|  | VCT |  |
|  | NHTW |  |
|  | Man up |  |
|  | Man-up |  |
|  | #knowingissexy |  |
|  | *Removed*: *latest, greatest, protest, protests, contest, contests* |  |
| Harm reduction | Harm reduction | Syringe exchange programs not only save money, but they also save lives!  #NeedleExchange #OpioidEpidemic #HarmReduction #HIVprevention #Infectionprevention (2019) |
|  | Reducing harm |  |
|  | Substance abuse |  |
|  | Substance use |  |
|  | SUD |  |
|  | Injection drug use |  |
|  | Injecting drug users |  |
|  | Using drugs |  |
|  | Drug abuse |  |
|  | Inject |  |
|  | IDU |  |
|  | FIDU |  |
|  | PWID |  |
|  | PWUD |  |
|  | Needle |  |
|  | Syringe |  |
|  | NSEP |  |
|  | NEP |  |
|  | SEP |  |
|  | MOUD |  |
|  | Chemsex |  |
|  | Alcohol |  |
|  | Drinking |  |
|  | Drinks |  |
|  | Addiction |  |
|  | Snow blow |  |
|  | Opiate |  |
|  | Opioid |  |
|  | Methadone |  |
|  | Buprenorphine |  |
|  | Bupe |  |
|  | #saynotodrugs |  |
|  | #safeinjecting |  |
|  | #saferinjecting |  |
| Gender inequity and violence against women | Violence against women | A. Addressing the chronic, global violence against women is #HIVprevention for women #StepUp4Women (2014) |
|  | VAW |  |
|  | Gender-based violence |  |
|  | Gender based violence |  |
|  | GBV |  |
|  | Rape |  |
|  | Sexual violence |  |
|  | Sexual assault |  |
|  | Sexual harassment |  |
|  | Sexual exploitation |  |
|  | Domestic violence |  |
|  | Intimate partner violence |  |
|  | IPV |  |
|  | Human trafficking |  |
|  | Child marriage |  |
|  | Female genital mutilation |  |
|  | FGM |  |
|  | Gender based violence |  |
|  | Gender-based violence |  |
|  | Gender norms |  |
|  | Gender equality |  |
|  | Gender inequality |  |
|  | Gender equity |  |
|  | Gender inequity |  |
|  | Gender health equality |  |
|  | Gender health inequality |  |
|  | Gender health equity |  |
|  | Gender health inequity |  |
|  | #StepUp4Women |  |
|  | #orangetheworld |  |
|  | #girlscount |  |
|  | #ProtectWomen |  |
|  | #WhatWomenWant |  |
|  | #WhatGirlsWant |  |
| Sex work | Sex work | #HIVprevention programs for sex workers should focus on reducing stigma that communities attach to sex work thus creating safer environment  (2016) |
|  | Transactional sex |  |
|  | Commercial sex |  |
|  | Sex trade |  |
|  | FSW |  |
|  | Exchange sex |  |
|  | Prostitution |  |
|  | Prostitute |  |
| Uncategorized^d^ | N/A | Everyone has a right to #HIVprevention &amp; be able to access #prevention methods that fit their lives. Hands up for #HIVprevention #WAD2016 (2016) |

^a^Pattern matching based on keywords detected the specified word and all of its children (e.g., ‘test’ captured ‘tests’ and ‘testing’). The keywords were not case-sensitive (e.g., ‘EMTCT’ also included references to ‘emtct’). A space was included after some words so as to intentionally avoid the capture of larger words (e.g., SUD did not also capture ‘suddenly’).

^b^For each keyword listed above, if not already a hashtag keyword, the hashtag equivalent of the keyword was also included in the pattern matching procedure (e.g., ‘Wrap it up’ and ‘#wrapitup’) were included. For the sake of space, these hashtag equivalents were not presented in the table.

^c^Tweets were modified for the table by removing links, emojis, names and Twitter handles.

^d^Uncategorized tweets includes all tweets that did not contain at least one of the keywords listed in the table.
